# Noval soliton solution, sensitivity and stability analysis to the fractional gKdV-ZK equation

**DOI:** 10.1038/s41598-024-51577-8

**Published:** 2024-02-14

**Authors:** Muhammad Shakeel, Asim Zafar, Abdu Alameri, Muhammad Junaid U Rehman, Jan Awrejcewicz, Muhammad Umer, Muhammad Zahid, Kottakkaran Sooppy Nisar

**Affiliations:** 1 School of Mathematics and Statistics, Central South University, Changsha, 410083 China; 2 Department of Mathematics, COMSATS University, Vehari Campus, Islamabad, Pakistan; 3 Department of Biomedical Engineering, University of Science and Technology, Sana’a, Yemen; 4 Department of Automation, Biomechanics, and Mechatronics, Lodz University of Technology, 1/15 Stefanowski St. (Building A22), Lodz, 90-924 Poland; 5 Institute of Turbomachinery, Lodz University of Technology, Wólczanska 219/221, 90-924 Lodz, Poland; 6 Department of Mathematics, College of Arts and Sciences, Prince Sattam Bin Abdulaziz University, Wadi Aldawaser, Saudi Arabia

**Keywords:** Mathematics and computing, Physics

## Abstract

This work examines the fractional generalized Korteweg-de-Vries-Zakharov-Kuznetsov equation (gKdV-ZKe) by utilizing three well-known analytical methods, the modified $\left( \frac{G^{'}}{G^2}\right)$-expansion method, $\left( \frac{1}{G^{'}}\right)$-expansion method and the Kudryashov method. The gKdV-ZK equation is a nonlinear model describing the influence of magnetic field on weak ion-acoustic waves in plasma made up of cool and hot electrons. The kink, singular, anti-kink, periodic, and bright soliton solutions are observed. The effect of the fractional parameter on wave shapes have been analyzed by displaying various graphs for fractional-order values of $\beta$. In addition, we utilize the Hamiltonian property to observe the stability of the attained solution and Galilean transformation for sensitivity analysis. The suggested methods can also be utilized to evaluate the nonlinear models that are being developed in a variety of scientific and technological fields, such as plasma physics. Findings show the effectiveness simplicity, and generalizability of the chosen computational approach, even when applied to complex models.

## Introduction

Fractional Partial differential equations (FPDEs) can be considered as the generalized type of partial differential equations (PDEs). The FDEs have attracted the researchers’ attention over the past two decades because the results of PDEs are neglected. The search for the exact solutions of FPDEs plays a vital role in understanding the qualitative and quantitative features of many physical phenomena, which are expressed by these equations^1–6^. For instance, the nonlinear oscillation of an earthquake can be modeled by derivatives of fractional order. The physical phenomena may not depend only on the time moment but also on the former time history, which can be successfully modeled utilizing the theory of fractional integrals and derivatives.

Nonlinear fractional partial differential equations (NFPDEs) have a significant role in various fields like applied mathematics, optical fiber, engineering, fluid, wave motion, mechanics, and plasma physics; they produce an essential part of the modelling of real-world issues. Nowadays, analytical solutions are becoming more important in various engineering and mathematics fields. The prominent investigators of this era are more interested in producing novel solutions for different.

Recently many powerful techniques for attaining the exact solution of NPDEs have been presented, such as Jacobi-elliptic approach^7^, Sine-Gordon expansion scheme^8–10^, modified simple equation scheme^11^, the Kudryashov approach^12^, auxiliary equation technique^13,14^, Exp-function method^15^, the extended direct algebraic method^16–19^, $\left( \frac{G^{'}}{G^{2}}\right)$-expansion method^17,20^, extended tanh expansion scheme^21^, $(m+\frac{G^{'}}{G})$-expansion method^22^, Hirota bilinear method^23,24^, modified rational expansion method^25^, modified Sardar sub-equation method^26^, the Riccati equation mapping method^27^, F-expansion method^28^ and many more^29–33^.

In this paper, an effective method like modified $\left( \frac{G^{'}}{G^2}\right)$-expansion method, modified $\left( \frac{1}{G^{'}}\right)$- expansion method, and the Kudryashov method is utilized for investigating a variety of soliton solutions for gKdV-ZK fractional equation. This equation is used in plasma physics for analyzing the ion-acoustic wave structures^34,35^.

1 $$\begin{aligned} \frac{\partial ^\beta u}{\partial t^\beta }+a u^2 \frac{\partial u}{\partial x}+b \frac{\partial ^3 u}{\partial x^3}+ d \frac{\partial }{\partial x}\left( \frac{\partial ^2 u}{\partial y^2}+\frac{\partial ^2 u}{\partial z^2}\right) =0, \end{aligned}$$

where *a*,*b*, and *d* are the constants. The gKdV-ZK fractional equation is a special type of nonlinear evolution equation that can be used to describe different complex nonlinear phenomena in the various fields of nonlinear science such as , plasma physics, fluid dynamics, and electromagnetism. The analytical solutions of (1) were attained by utilizing Kudryashov’s technique, and Jacobi elliptic function scheme^36^. The hot isothermal and warm adiabatic fluid mixtures were derived in^37^. The electron acoustic solitons for a small amplitude region were investigated in^38^. The exact solutions of Eq. (1) were attained by utilizing Kudryashov’s technique, and Jacobi elliptic function scheme^36^. The kink, quasi-periodic and lump-type soliton of Eq. (1) were acquired by utilizing the Lie symmetry approuch^39^. In the past modified $\left( \frac{G^{'}}{G^2}\right)$-expansion technique, $\left( \frac{1}{G^{'}}\right)$-expansion approach and the Kudryashov scheme were used on different equation such as: In^40^ the variety of traveling solution was obtained. In^41^, the analytical solutions for Gardner equations were achieved by utilizing $\left( \frac{1}{G^{'}}\right)$-expansion technique. By utilizing the modified $\left( \frac{G^{'}}{G^2}\right)$-expansion approach, the traveling wave solutions were obtained for the nonlinear Schrodinger equation in^42^. The soliton solutions of the Fokas-Lenells model also have been attained by utilizing $\left( \frac{G^{'}}{G^{2}}\right)$-expansion approach^43^. The topological, periodic, and singular soliton solutions were attained in^44^ by utilizing the Kudryashov method. The soliton solutions of the Maccari equation were investigated with the aid of the Kudryashov scheme^45^. Different definitions for fractional derivatives have been utilized in the last many years. Such as, Beta time-fractional^46^, Reimann-Liouville^47^, Caputo fractional^48^, Conformable fractional^49^, truncated M-fractional derivative^50^.

This research work is divided into sections: In section(2) we described the Beta derivative. In section(3) modified $\left( \frac{G^{'}}{G^2}\right)$-expansion method is utilized on Eq. (2) to attained the periodic and singular type soliton . The kink and dark type soliton are retrieved by using $\left( \frac{1}{G^{'}}\right)$-expansion method in section(4). Section (5) discussed the Kudryashov scheme. The sensitivity and stability analysis of the soliton solution is discussed in section(6). In section(7) graphically representation. In the end, the conclusion is presented in section(8).

## Beta derivative

### Definition:

 Let *P*(*t*) be a function defined $\forall$ non-negative *t*. Then, the $\beta$ derivative of *P*(*t*) of order $\beta$ is given by^51^

$$\begin{aligned} T^\beta p(t)=\frac{d^\beta p(t)}{dt^\beta }=\lim \nolimits _{\epsilon \rightarrow 0}{\frac{p(t+\epsilon (t+\frac{1}{\Gamma (\beta )})^{1-\beta })-p(t)}{\epsilon }},\,\,0<\beta \le 1 \end{aligned}$$

### Remark:

$$\begin{aligned} T^\beta (p(t))=\left( t+\frac{1}{\Gamma (\beta )}\right) ^{1-\beta } \frac{dp(t)}{dt} \end{aligned},$$

where $$t>0$$ and $$\beta \in (0,1]$$.

## The modified $\left( \frac{G^{'}}{G^2}\right)$-expansion method

Consider the NPDE is

2 $$\begin{aligned} \Theta (u, D_t u,D_x u, D^2_t u,...)=0, \end{aligned}$$

where operator *D* represents the partial derivative and *u* is an unknown function.

Consider the travelling wave is

3 $$\begin{aligned} u(x,t)=U(\eta ),\,\,\,\, \eta =x+y+z-\frac{c}{\beta }\left( t+\frac{1}{\Gamma (\beta )}\right) ^{\beta }, \end{aligned}$$

utilizing (3) into (2),then

4 $$\begin{aligned} \digamma (U, U^{'}, U^{''}, U^{'''},...)=0. \end{aligned}$$

The travelling wave solutions are

5 $$\begin{aligned} U(\eta )=\sum _{n=0}^{N}b_n\left( \frac{G^{'}}{G^2}\right) ^n,\,\, where \,\, n=1,2,3,...,N \end{aligned}$$

6 $$\begin{aligned} \left( \frac{G^{'}}{G^2}\right) ^{'}=\sigma _1+\tau _1 \left( \frac{G^{'}}{G^2}\right) ^2, \end{aligned}$$

where $\tau _1, \sigma _1$ and $b_n$ are unknown parameters which find latter.

The (6) has three cases:

### Case-1

 If $$\sigma _1\tau _1>0$$,

7 $$\begin{aligned} \left( \frac{G^{'}}{G^2}\right) =\sqrt{\frac{\sigma _1}{\tau _1}}\left( \frac{A_1\cos \sqrt{\sigma _1\tau _1}\eta +B_1\sin \sqrt{\sigma _1\tau _1}\eta }{A_1\sin \sqrt{\sigma _1\tau _1} \eta -B_1\cos \sqrt{\sigma _1\tau _1}\eta }\right) , \end{aligned}$$

where $A_1$ and $B_1$ are arbitrary nonzero constants.

### Case-2

 If $$\sigma _1\tau _1<0$$,

8 $$\begin{aligned} \left( \frac{G^{'}}{G^2}\right) =-\frac{\sqrt{\mid \sigma _1\tau _1\mid }}{\tau _1}+\frac{\sqrt{\mid \sigma _1\tau _1\mid }}{2}\left( \frac{A_1\sinh (2\sqrt{\mid \sigma _1\tau _1\mid }\eta )+B_1\cosh (2\sqrt{\mid \sigma _1\tau _1\mid }\eta )}{A_1 \cosh (2\sqrt{\mid \sigma _1\tau _1\mid }\eta )+B_1\sinh (2\sqrt{\mid \sigma _1\tau _1\mid }\eta )}\right) . \end{aligned}$$

### Case-3

 If $$\sigma _1=0, \tau _1\ne 0$$,

9 $$\begin{aligned} \left( \frac{G^{'}}{G^2}\right) =-\frac{A_1}{\tau _1(A_1\eta +B_1)}. \end{aligned}$$

To obtain the three types of solution by putting the value of unknown $b_n$ and Eqs. (7),(8),(9) into (5).

### Application of modified $\left( \frac{G^{'}}{G^2}\right)$-expansion method

The gKdV-ZKe equation is,

10 $$\begin{aligned} \frac{\partial ^\beta u}{\partial t^\beta }+a u^2 \frac{\partial u}{\partial x}+b \frac{\partial ^3 u}{\partial x^3}+ d \frac{\partial }{\partial x}\left( \frac{\partial ^2 u}{\partial y^2}+\frac{\partial ^2 u}{\partial z^2}\right) =0, \end{aligned}$$

Suppose the transformation,

11 $$\begin{aligned} u(x,y,z,t)=U(\eta ),\, \eta =x+y+z-\frac{c}{\beta }(t+\frac{1}{\Gamma (\beta )})^{\beta }, \end{aligned}$$

on (10), we get

12 $$\begin{aligned} -cU^{'}+aU^2U^{'}+bU^{'''}+2 d U^{''}=0. \end{aligned}$$

Integrate (12) one time with respect to $\eta$, we get

13 $$\begin{aligned} -cU+a\frac{U^3}{3}+(b+2d)U^{''}=0. \end{aligned}$$

Utilizing the homogenous balance approach on (10), then we have $N=1$,

14 $$\begin{aligned} U(\eta )=b_0+b_1\left( \frac{G^{'}}{G^2}\right) . \end{aligned}$$

Utilizing (14) into (13), then we get,

- $$\left( \frac{G^{'}}{G^2}\right) ^0:\,\,\,\, \frac{b_1 b_0^3}{3}-b_0 c=0$$
- $$\left( \frac{G^{'}}{G^2}\right) ^1:\,\,\,\, a b_1 b_0^2+2 b_1 b \tau _1 \sigma _1 -b_1 c+4 b_1 \tau _1 \sigma _1 d=0$$
- $$\left( \frac{G^{'}}{G^2}\right) ^2:\,\,\,\,a b_0 b_1^2=0$$
- $$\left( \frac{G^{'}}{G^2}\right) ^3:\,\,\,\, \frac{a b_1^3}{3}+2 b_1 b \tau _1 ^2+4 b_1 \tau _1 ^2 d=0$$

The solution of the above system is given below,

#### Set-1

15 $$\begin{aligned} b_0= 0, b_1=\frac{i \sqrt{3} \sqrt{c} \sqrt{\tau _1 }}{\sqrt{a } \sqrt{\sigma _1 }}, d =\frac{c-2 b \tau _1 \sigma _1 }{4 \tau _1 \sigma _1 }. \end{aligned}$$

(14) become,

16 $$\begin{aligned} U(\eta )=\left( \frac{i \sqrt{3} \sqrt{c} \sqrt{\tau _1 }}{\sqrt{a } \sqrt{\sigma _1 }}\right) \left( \frac{G^{'}}{G^2}\right) . \end{aligned}$$

Three different solutions are given below,

#### Case-1

 If $$\sigma _1\tau _1>0$$,

17 $$\begin{aligned} U(\eta )=\left( \frac{i \sqrt{3} \sqrt{c} \sqrt{\tau _1 }}{\sqrt{a } \sqrt{\sigma _1 }}\right) \left( \sqrt{\frac{\sigma _1}{\tau _1}}(\frac{A_1\cos \sqrt{\sigma _1\tau _1}\eta +B_1\sin \sqrt{\sigma _1\tau _1}\eta }{A_1\sin \sqrt{\sigma _1\tau _1} \eta -B_1\cos \sqrt{\sigma _1\tau _1}\eta })\right) . \end{aligned}$$

Where $$\eta =x+y+z-\frac{c}{\beta }(t+\frac{1}{\Gamma (\beta )})^{\beta }$$,

#### Case-2

 If $$\sigma _1\tau _1<0$$,

18 $$\begin{aligned} U(\eta )=\left( \frac{i \sqrt{3} \sqrt{c} \sqrt{\tau _1 }}{\sqrt{a } \sqrt{\sigma _1 }}\right) \left( -\frac{\sqrt{\mid \sigma _1\tau _1\mid }}{\tau _1}+\frac{\sqrt{\mid \sigma _1\tau _1\mid }}{2}(\frac{A_1\sinh (2\sqrt{\mid \sigma _1\tau _1\mid }\eta )+B_1\cosh (2\sqrt{\mid \sigma _1\tau _1\mid }\eta )}{A_1 \cosh (2\sqrt{\mid \sigma _1\tau _1\mid }\eta )+B_1\sinh (2\sqrt{\mid \sigma _1\tau _1\mid }\eta )}\right) . \end{aligned}$$

#### Case-3

If $$\sigma _1=0, \tau _1\ne 0$$,

19 $$\begin{aligned} U(\eta )=\left( \frac{i \sqrt{3} \sqrt{c} \sqrt{\tau _1 }}{\sqrt{a } \sqrt{\sigma _1 }}\right) \left( -\frac{A_1}{\tau _1(A_1\eta +B_1)}\right) . \end{aligned}$$

#### Set-2

20 $$\begin{aligned} b_0= 0, b_1=-\frac{i \sqrt{3} \sqrt{c} \sqrt{\tau _1 }}{\sqrt{a } \sqrt{\sigma _1 }}, d =\frac{c-2 b \tau _1 \sigma _1 }{4 \tau _1 \sigma _1 }. \end{aligned}$$

Equation (14) become,

21 $$\begin{aligned} U(\eta )=\left( -\frac{i \sqrt{3} \sqrt{c} \sqrt{\tau _1 }}{\sqrt{a } \sqrt{\sigma _1 }}\right) \left( \frac{G^{'}}{G^2}\right) . \end{aligned}$$

Three different solutions are given below,

#### Case-1

 If $$\sigma _1\tau _1>0$$,

22 $$\begin{aligned} U(\eta )=\left( -\frac{i \sqrt{3} \sqrt{c} \sqrt{\tau _1 }}{\sqrt{a } \sqrt{\sigma _1 }}\right) \left( \sqrt{\frac{\sigma _1}{\tau _1}}(\frac{A_1\cos \sqrt{\sigma _1\tau _1}\eta +B_1\sin \sqrt{\sigma _1\tau _1}\eta }{A_1\sin \sqrt{\sigma _1\tau _1} \eta -B_1\cos \sqrt{\sigma _1\tau _1}\eta })\right) . \end{aligned}$$

#### Case-2

 If $$\sigma _1\tau _1<0$$,

23 $$\begin{aligned} U(\eta )=\left( -\frac{i \sqrt{3} \sqrt{c} \sqrt{\tau _1 }}{\sqrt{a } \sqrt{\sigma _1 }}\right) \left( -\frac{\sqrt{\mid \sigma _1\tau _1\mid }}{\tau _1}+\frac{\sqrt{\mid \sigma _1\tau _1\mid }}{2}(\frac{A_1\sinh (2\sqrt{\mid \sigma _1\tau _1\mid }\eta )+B_1\cosh (2\sqrt{\mid \sigma _1\tau _1\mid }\eta )}{A_1 \cosh (2\sqrt{\mid \sigma _1\tau _1\mid }\eta )+B_1\sinh (2\sqrt{\mid \sigma _1\tau _1\mid }\eta )}\right) . \end{aligned}$$

#### Case-3

If $$\sigma _1=0, \tau _1\ne 0$$,

24 $$\begin{aligned} U(\eta )=\left( -\frac{i \sqrt{3} \sqrt{c} \sqrt{\tau _1 }}{\sqrt{a } \sqrt{\sigma _1 }}\right) \left( -\frac{A_1}{\tau _1(A_1\eta +B_1)}\right) . \end{aligned}$$

## The $\left( \frac{1}{G^{'}}\right)$-expansion method

Consider the Eqs. (2), (3), (4). The solution of (4) is,

25 $$\begin{aligned} U(\eta )=\sum _{n=0}^{N}b_n\left( \frac{1}{G^{'}}\right) ^n. \end{aligned}$$

The second order ODE is,

26 $$\begin{aligned} G^{''}(\eta )+\sigma _1 G^{'}(\eta )+\tau _1=0, \end{aligned}$$

where $a_n$, $\sigma _1$ and $\tau _1$ are unknown parameters to be determined later and *N* is homogenous balance number. The (26) become,

27 $$\begin{aligned} G(\eta )=A_1 e^{-\sigma _1\eta }-\frac{\tau _1}{\sigma _1}+A_2. \end{aligned}$$

Then,

28 $$\begin{aligned} \left( \frac{1}{G^{'}}\right) =\frac{\sigma _1}{-\tau _1+\sigma _1 A_1\left( \cosh (\sigma _1\eta )-\sinh (\sigma _1\eta )\right) }. \end{aligned}$$

Here, $A_1$ and $A_2$ are unknown parameters. Putting (25) into (4) and utilizing (26), then (4) can be changed into a polynomials of $(\frac{1}{G^{'}})$. After this, we are setting the polynomial equal to zero, and then we get a system of algebraic equations. Solving the obtained system with the aid of Mathematica to attain the values of parameters.

### Application of $\left( \frac{1}{G^{'}}\right)$-expansion method

Utilizing $N=1$ into (25), then we have

29 $$\begin{aligned} U(\eta )=b_0+b_1\left( \frac{G^{'}}{G^2}\right) , \end{aligned}$$

utilizing Eq. (29) into the Eq. (13) then we get set of algebraic equations

- $$\left( \frac{1}{G^{'}}\right) ^0:\,\,\,\frac{a b_0^3}{3}-b_0 c=0$$
- $$\left( \frac{1}{G^{'}}\right) ^1:\,\,\,\frac{a b_1^3}{3}+4 b_1 d \tau _1 ^2+2 b b_1 \tau _1 ^2=0$$
- $$\left( \frac{1}{G^{'}}\right) ^2:\,\,\, a b_0 b_1^2+6 b_1 d \sigma _1 \tau _1 +3 b b_1 \sigma _1 \tau _1 =0$$
- $$\left( \frac{1}{G^{'}}\right) ^3:\,\,\,a b_0^2 b_1-b_1 c+2 b_1 d \sigma _1 ^2+b b_1 \sigma _1 ^2=0$$

Solving the overhead system of the equation we acquire the solutions,

#### Set-1

30 $$\begin{aligned} b_0=\frac{\sqrt{3} \sqrt{c}}{\sqrt{a}},b_1=\frac{2 \sqrt{3} \sqrt{c} \tau _1 }{\sqrt{a} \sigma _1 },d=\frac{-b \sigma _1 ^2-2 c}{2 \sigma _1 ^2}. \end{aligned}$$

Putting (30) into (29), then solution of (1) is,

31 $$\begin{aligned} U(\eta )=\frac{\sqrt{3} \sqrt{c}}{\sqrt{a}}+\frac{2 \sqrt{3} \sqrt{c} \tau _1 }{\sqrt{a} \sigma _1 }\left( \frac{\sigma _1}{-\tau _1+\sigma _1 A_1\left( \cosh (\sigma _1\eta )-\sinh (\sigma _1\eta )\right) }\right) , \end{aligned}$$

#### Set-2

32 $$\begin{aligned} b_0=-\frac{\sqrt{3} \sqrt{c}}{\sqrt{a}},b_1=-\frac{2 \sqrt{3} \sqrt{c} \tau _1 }{\sqrt{a} \sigma _1 },d=\frac{-b \sigma _1 ^2-2 c}{2 \sigma _1 ^2}. \end{aligned}$$

Putting (34) into (29), then solution of (1) is,

33 $$\begin{aligned} U(\eta )=-\frac{\sqrt{3} \sqrt{c}}{\sqrt{a}}-\frac{2 \sqrt{3} \sqrt{c} \tau _1 }{\sqrt{a} \sigma _1 }\left( \frac{\sigma _1}{-\tau _1+\sigma _1 A_1\left( \cosh (\sigma _1\eta )-\sinh (\sigma _1\eta )\right) }\right) . \end{aligned}$$

## Kudryashov method

Solution of Eq. (4) is,

34 $$\begin{aligned} U(\eta )=b_0+b_1Q(\eta )+...+b_N Q(\eta )^N, b_N\ne 0, \end{aligned}$$

where $b_i$ is unknown, *N* is homogenous balance number, and $Q(\eta )$ is the solution,

$$Q(\eta )^2=\gamma ^2 R(\eta )^2(1-\rho Q(\eta )^2)$$, $$Q(\eta )=\frac{4 \kappa }{4\kappa ^2e^{\gamma \eta }+\rho e^{-\gamma \eta }}$$,

Now putting (34) into (12) and obtaining the algebraic system by solving the system we lead soliton solution of the NPDE Eq. (1).

### Application of Kudryashov method

Substituting $N=1$ into (34) then,

35 $$\begin{aligned} U(\eta )=b_0+b_1Q(\eta ). \end{aligned}$$

Putting (29) into (13) then we get set of algebraic equations

- $$Q(\eta )^{0}:\,\,\frac{a b_0^3}{3}-b_0 c=0$$
- $$Q(\eta )^{1}:\,\,a b_0^2 b_1+b b_1 \gamma ^2-b_1 c+2 b_1 \gamma ^2 d=0$$
- $$Q(\eta )^{2}:\,\,a b_0 b_1^2=0$$
- $$Q(\eta )^{3}:\,\,\frac{a b_1^3}{3}-2 b b_1 \gamma ^2 \rho -4 b_1 \gamma ^2 d \rho =0$$

Resolving the above system of equations we get the following solutions,

#### Set-1

36 $$\begin{aligned} b_0= 0,b_1=-\frac{\sqrt{6} \sqrt{c} \sqrt{\rho }}{\sqrt{a}},d=\frac{c-b \gamma ^2}{2 \gamma ^2}. \end{aligned}$$

Putting (36) into (35), then solution of equation (1) is,

37 $$\begin{aligned} U(\eta )=\left( -\frac{\sqrt{6} \sqrt{c} \sqrt{\rho }}{\sqrt{a}}\right) \left( \frac{4 \kappa }{4\kappa ^2e^{\gamma \eta }+\rho e^{-\gamma \eta }}\right) . \end{aligned}$$

#### Set-2

38 $$\begin{aligned} b_0= & {} 0,b_1=\frac{\sqrt{6} \sqrt{c} \sqrt{\rho }}{\sqrt{a}},d=\frac{c-b \gamma ^2}{2 \gamma ^2}. \end{aligned}$$

39 $$\begin{aligned} U(\eta )= & {} \left( \frac{\sqrt{6} \sqrt{c} \sqrt{\rho }}{\sqrt{a}}\right) \left( \frac{4 \kappa }{4\kappa ^2e^{\gamma \eta }+\rho e^{-\gamma \eta }}\right) . \end{aligned}$$

### Sensitivity analysis

From (11), we can write as

40 $$\begin{aligned} U^{''}=\frac{c}{b+2d}U -\frac{a}{b+2d}U^3. \end{aligned}$$

Let $\frac{c}{b+2d}=A$ and $\frac{a}{b+2d}=B$ then we get,

41 $$\begin{aligned} U^{''}=AU-BU^3. \end{aligned}$$

Using the Galilean transformation on (41) then we get dynamical system as:

42 $$\begin{aligned} {\left\{ \begin{array}{ll} U^{'}=H, \\ H^{'}=AU-BU^3. \end{array}\right. } \end{aligned}$$

We will now investigate the sensitive phenomena of the perturbed system shown below. Subsequently, we will decompose the schemes given in Eq. (42) into an autonomous conservative dynamical system (ACDS), as illustrated below:

43 $$\begin{aligned} {\left\{ \begin{array}{ll} U^{'}=H, \\ H^{'}=AU-BU^3+m_{0}\cos (f\eta ). \end{array}\right. } \end{aligned}$$

**Figure 1:**
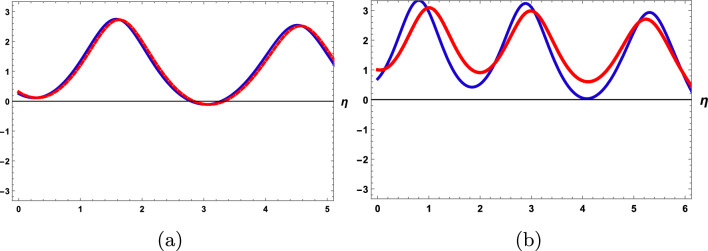
Sensitivity behaviour of the perturbed system (43) letting the initial condition (**a**) (0, 0.25) for blue solid line and (0.01, 0.30) for red dotted curve, (**b**) (0.05,0.8) for blue solid line and (0.08,1).

In which *f* represents to be the frequency and $m_0$ is the strength of the perturbed component^52^. In the current part of the investigation, we will explore whether the frequency term has any effect on the model which will be examined. To do this, we will evaluate the model under examination’s particular appearance and address the impact of the perturbation’s force and frequency. By using four different beginning conditions in the component, we aim to evaluate the sensitivity of such a solution to the perturbed dynamical structural Eq. (43) at the value of parameters $c=0.05, a=0.5,b=d=f=0.2,m_0=4.5.$ From Fig. 1 we have seen that In Fig(a), the system is not sensitive because there is overlapping in the cure but with a small change in the initial condition system becomes sensitive.

### Stability analysis

The stability of the solitary wave solution is discussed in this section with the help of the Hamiltonian system. The HSM condition is given by^53^,

44 $$\begin{aligned} M_1=\frac{1}{2}\int _{a_{1}}^{a_{2}}{U(\eta )} d\eta , \end{aligned}$$

**Figure 2:**
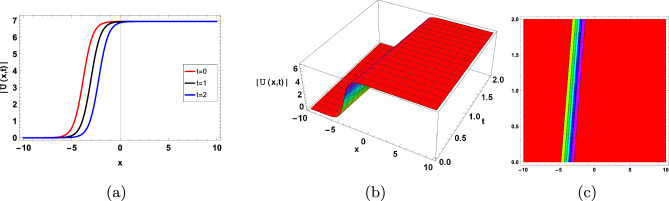
$3-D, 2-D,$ and contour type solitary graph of (33).

**Figure 3:**
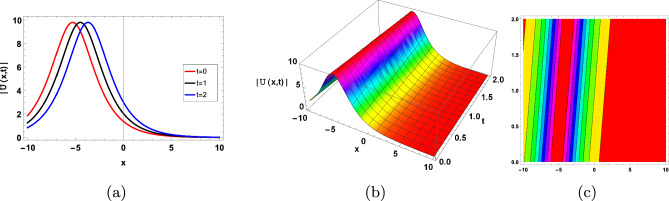
$3-D, 2-D,$ and contour type solitary representation of (37).

Here, *U* represent the dependent variable, $a_{1}$, $a_{2}$ are arbitrary constants and satisfies $a_{1}<a_{2}$. The following criteria determine how dependent the stability of the obtained solutions is on the HSM:

45 $$\begin{aligned} \frac{\partial M_1}{\partial c}>0, \end{aligned}$$

where *c* is the speed of waves. The selected values for parameter is given by $(g_1=0.1,\nu =-0.8, g_3=0.3, \tau =0.05, y=0.5, g_2=0.08, z=0.5, \varsigma =0.1)$ make the (33) and (37) stable solution as shown in Fig.(2,3) when $t\in [0,2]$, and $x\in [1,10]$. We utilized the same steps for the other soliton solutions to check their stability property.

## Results and discussion

**Figure 4:**
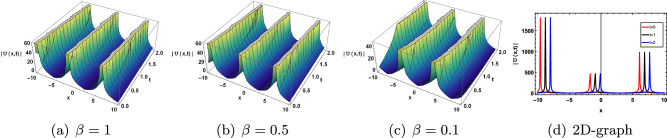
Effect of parameter $\beta$ on (17).

**Figure 5:**
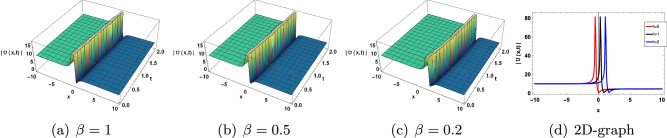
Effect of parameter $\beta$ on (18).

**Figure 6:**
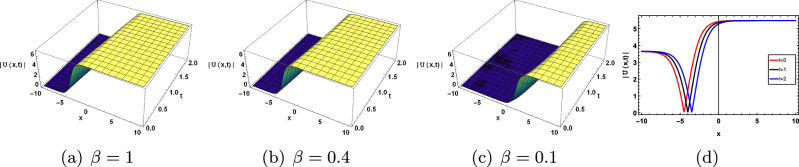
Effect of parameter $\beta$ on (30).

**Figure 7:**
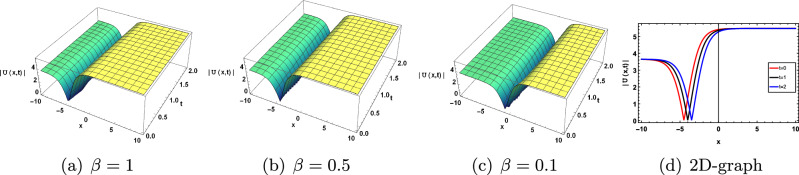
Effect of parameter $\beta$ on (33).

**Figure 8:**
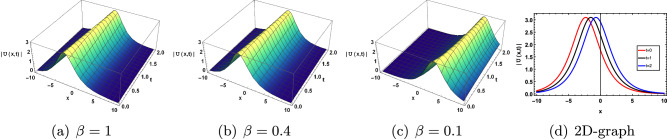
Effect of parameter $\beta$ on (37).

This section discusses the graphical presentation of the gKdV-ZK equation. The physical phenomena of the nonlinear model are determined by giving suitable values to the arbitrary constants with the help of Mathematica. We illustrate 2 and 3-dimensional Figs. 4, 5, 6, 7 and 8 of some obtained solutions to best analyze the nature of solitary wave solution. 3D and 2D shape of the solution (17) are presented in Fig. 4. Figure 4$(a)-(c)$ show the periodic type wave profile of (17) for choosing the parameteric values $c=0.8, b=2, a=-0.05, \sigma _1 =2,\tau _1 =0.08, k=-0.05, y=5, z=-0.5 , A_1=0.05, B_1=0.05$ within the range $-10\le x\le 10$ and $0\le t \le 2$. Different wave structure for diverse values of $\beta$ is present in Fig.(4)$(a)-(c)$. 2D graph with respect to time t is presented in Fig. 4d. We have also observed that the solitary waves tiny shifts when the change fractional order beta is without changing the shape of the curve. Furthermore, we have compared our solutions with Romana et al.^54^ that have attained bright and single soliton forms with the aid of an improved modified extended tanh expansion method (METEM). But in this article, we have achieved different forms such as bright, dark, singular, kink and anti-kink of soliton solutions that have applications in plasma physics. Comparison with the solution of the METE method is shown in Table 1.

**Table 1:** Results of Different Methods.

Modified $\left( \frac{G^{'}}{G^2}\right)$-expansion method	
$$b_0= 0, b_1=\pm \frac{i \sqrt{3} \sqrt{c} \sqrt{\tau _1 }}{\sqrt{a } \sqrt{\sigma _1 }}, d =\frac{c-2 b \tau _1 \sigma _1 }{4 \tau _1 \sigma _1 }$$	
$\left( \frac{1}{G^{'}}\right)$-expansion method	
$$b_0=\frac{\sqrt{3} \sqrt{c}}{\sqrt{a}},b_1=\pm \frac{2 \sqrt{3} \sqrt{c} \tau _1 }{\sqrt{a} \sigma _1 },d=\frac{-b \sigma _1 ^2-2 c}{2 \sigma _1 ^2}$$	
Kudryashov method	
$$b_0= 0, b_1=\pm \frac{i \sqrt{3} \sqrt{c} \sqrt{\tau _1 }}{\sqrt{a } \sqrt{\sigma _1 }}, d =\frac{c-2 b \tau _1 \sigma _1 }{4 \tau _1 \sigma _1 }$$	
METE method	
$$b_0=\pm \frac{\sqrt{3} \sqrt{c}}{\sqrt{a}},b_1=-\frac{2 \sqrt{3} \sqrt{c} \tau _1 }{\sqrt{a} \sigma _1 },d=\frac{-b \sigma _1 ^2-2 c}{2 \sigma _1 ^2}$$	

Figure 5 shows the cupson-singular type wave profile depicted from the solution of (18) choosing the various values of parameter $c=0.8, b=2, a=0.05, \sigma _1 =-0.2, \tau _1 =0.8, k=0.5, y=0.5,z=0.5, A_1=0.01, B_1=0.05$.

The solution of (30) shows the kink soliton solution for the distinct values of parameter $c=0.8, b=2, a=-0.05, \sigma =2, \tau =0.08, k=-0.05, y=5, z=-0.5$ which is shown in Fig. 6.

3*D* and 2*D* shape of the solution (33) are presented in Fig. 7. Figure 7$(a)-(c)$ show the dark type solution of (33) at distinct values of parameter $c=0.8, b=2, a=-0.05, \sigma _1 =2,\tau _1 =0.08, k=-0.05, y=5, z=-0.5 , A_1=0.05, B_1=0.05$.

The solution of (37) represents the bright soliton for the distinct values of parameter $c=0.8, a=0.5, \rho =0.2, b=0.1,\kappa =0.5, \gamma =0.5, y=1, z=0.5$ which is shown in Fig. 8.

## Conclusion

We have successfully analyzed the fractional effect on the gKdV-ZK equation. We have been applying the modified $\left( \frac{G^{'}}{G^2}\right)$-expansion method, $\left( \frac{1}{G^{'}}\right)$-expansion method and kudryashov method on the resultant ODE to attain the different type of soliton solution.We have observed that the solitary waves tiny shifts when the change fractional order beta is without changing the shape of the curve. These methods retrieved the bright, dark, kink, anti-kink, cupson-singular, and periodic soliton solution Figs. 4, 5, 6, 7 and 8. The soliton solution of (33) and (37) are stable without brakes or discontinuity in plotted figures because these solutions fulfil the requirements of (45). These techniques perform consistently and successfully. The results investigated in this paper are verified and described with the help of graphs. The finding is very helpful in the investigation of shallow-water waves, ionic acoustic waves in plasma, long internal waves in density-stratified oceans, and sound waves on the crystal network. Furthermore, these solutions are very fruitful for the study of dynamic systems.

## Data Availability

All data that support the findings of this study are included in the article.
